# A Solution-Based Temperature Sensor Using the Organic Compound CuTsPc

**DOI:** 10.3390/s140609878

**Published:** 2014-06-04

**Authors:** Shahino Mah Abdullah, Zubair Ahmad, Khaulah Sulaiman

**Affiliations:** Low Dimensional Materials Research Centre, Department of Physics, Faculty of Science, University of Malaya, 50603 Kuala Lumpur, Malaysia; E-Mails: shahinomah@gmail.com (S.M.A.); zubairtarar@gmail.com (Z.A.)

**Keywords:** electrochemical, temperature sensor, copper phthalocyanine

## Abstract

An electrochemical cell using an organic compound, copper (II) phthalocyanine-tetrasulfonic acid tetrasodium salt (CuTsPc,) has been fabricated and investigated as a solution-based temperature sensor. The capacitance and resistance of the ITO/CuTsPc solution/ITO chemical cell has been characterized as a function of temperature in the temperature range of 25–80 °C. A linear response with minimal hysteresis is observed. The fabricated temperature sensor has shown high consistency and sensitive response towards a specific range of temperature values.

## 1. Introduction

Temperature is a key physical quantity that must be taken into account in almost all areas of science-related studies [1]. A proper temperature measurement technique is crucial as temperature is one of the most frequently measured physical quantities in measurement science [2]. Recently, a number of studies on temperature sensors for physical and chemical measurements have been performed with the aim of searching for linear and consistent sensing devices [3,4]. The need for reliable, inexpensive and harmless temperature sensors that can be operated simply is also a concern for many consumers, research and industrial applications [5–9].

It is known that many electrochemical sensing devices are very sensitive towards a small change in temperature. Large numbers of electrochemical sensors are increasingly being produced due to advances in design, component selectivity, and microminiaturization fabrication [10]. For these reasons, electrochemical cells could be well suited as sensing devices for temperature measurement. The cells typically consist of an electrolyte solution contained within two metal electrodes and a passed through current through an ionic conduction mechanism with oxidation and reduction processes occurring at the respective cell electrodes under ambient conditions. [11]. It is reported that the temperature variations can affect a conductivity of the cells [12]. The temperature may cause thermally enhanced ionic transport to occur [13]. As the temperature increases, the ionic and thermal conduction also increase leading to the increase of the cell's capacitance but a decrease in its resistance. By using this principle, an electrochemical cell using organic phthalocyanine (Pc) solution as electrolyte and transparent conductive oxide (TCO) as electrodes and functioning as a temperature sensor is studied in this paper.

Our previous investigation on temperature-sensitive chemical cells using nickel (II) phthalocyanine-tetrasulfonic acid tetrasodium salt (NiTsPc) aqueous solution had shown good temperature dependency characteristics [14]. The capacitance and resistance of the cells changed almost linearly with respect to the temperature variation with little hysteresis. These devices also showed a faster response and recovery compared to common organic-based sensors. By considering the potential advantage of this material, in our new work the same class of organic compound, but a different metal complex, a copper (II) phthalocyanine-tetrasulfonic acid tetrasodium salt (CuTsPc) was used to develop an enhanced temperature sensor with higher sensitivity and consistent response in the temperature regime from room temperature to below 100 °C. In addition, the enhancement is believed to result from the dimensions of the container for electrolyte solution as well. For this investigation, a small scale chemical cell, which consists of two ITO-coated surfaces facing to each other designed as working electrodes, has been made. We show that the sensitivity of these cells as a function of temperature is greatly increased.

## 2. Materials and Methods

**Figure 1.**
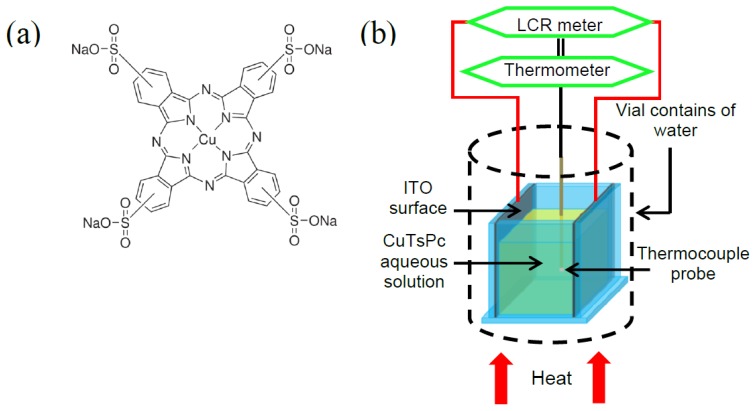
Molecular structure of copper (II) phthalocyanine-tetrasulfonic acid tetrasodium salt.

Copper (II) phthalocyanine-tetrasulfonic acid tetrasodium salt (CuTsPc) (988.28 g/mol molecular weight) was purchased from Sigma-Aldrich (St. Louis, MO, USA). Its molecular formula is C_32_H_16_CuN_8_O_12_S_4_Na_4_ and its molecular structure is as shown in Figure 1a. The CuTsPc aqueous solution has been prepared using de-ionized water at seven different weight percentages (wt.%) for composition optimization and selection. Firstly, the selection was made according to the maximum reach of both capacitance and resistance values with respect to the concentration difference. Secondly, it was confirmed by the minimum point of standard deviation of both capacitance and resistance of those concentrations. Then, the selection was made from these concentrations and a standard deviation plot corresponding to their resistance and capacitance. The sensor (chemical cell) container was fully fabricated using silica glass, in which two inner ITO-coated surfaces face each other at 7 mm distance. The area of each electrode was 140 mm^2^, actively working with 1 mL CuTsPc aqueous solution. The schematic diagram of the sensor is as shown in Figure 1b. Due to the small size of the chemical cell, the variation of solution temperature inside this container can only be measured using a thermocouple probe. The probe used in this experiment is Type-K thermocouple which can measure the temperature with the accuracy of ±1% in the range between −40 °C to 400 °C. It is used along with a Fluke 116 HVAC Multimeter. The cell was placed inside a vial that was filled with water and the whole cell was dipped in the water, except for the electrode connections region. This water was used as a medium to ensure the uniform distribution of heat from all sides of the cell. An LCR meter (GW Instek 829) was used to record the values of resistance and capacitance simultaneously by making a direct connection with the ITO electrodes. Both values were taken in steps of 5 °C in increasing and decreasing directions. The delay time to record the data at two different temperatures was 5 min in order to make sure that the heat was uniformly distributed in the all dimensions of the vial. The temperature was gradually controlled to observe the changes in capacitance and resistance accurately. The experiment has been done repeatedly in different ranges of applied temperature until a stable temperature regime was observed. The measurements were repeated several times in order to find the percentage error in the measurements, which been presented in the form of error bars as shown in the Discussion section.

## 3. Results and Discussions

**Figure 2.**
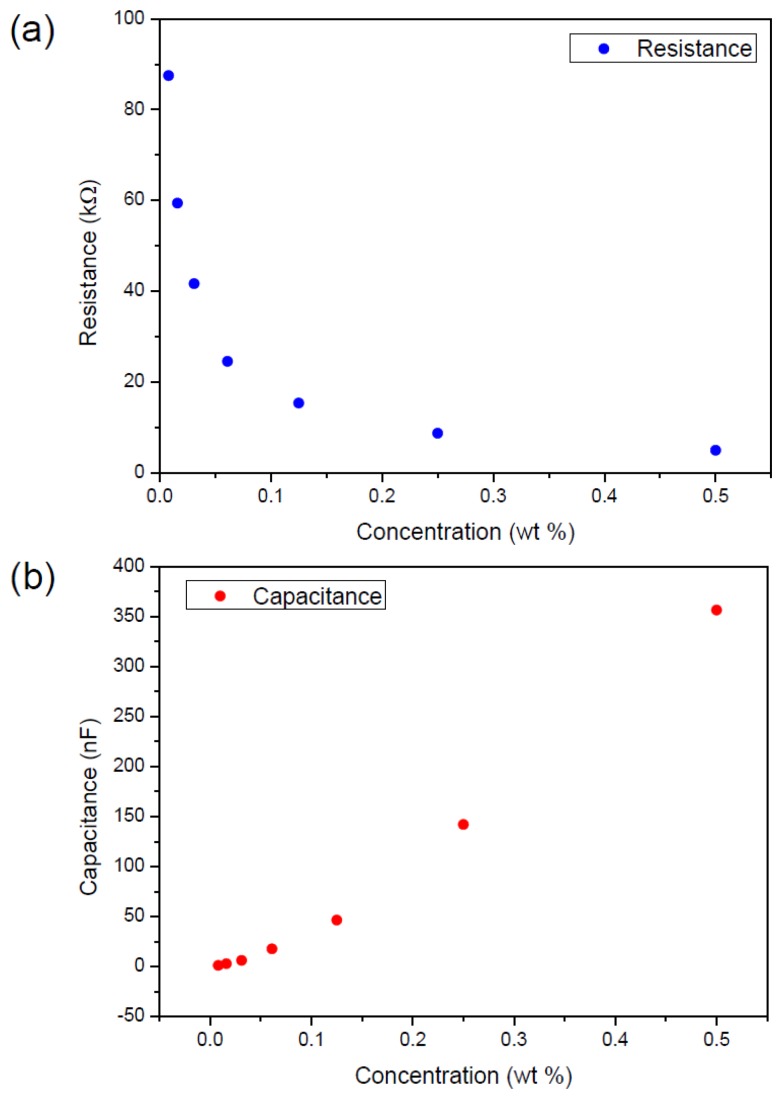
The effect of CuTsPc concentration on (**a**) resistance, and (**b**) capacitance of the ITO/CuTsPc solution/ITO cell.

**Figure 3.**
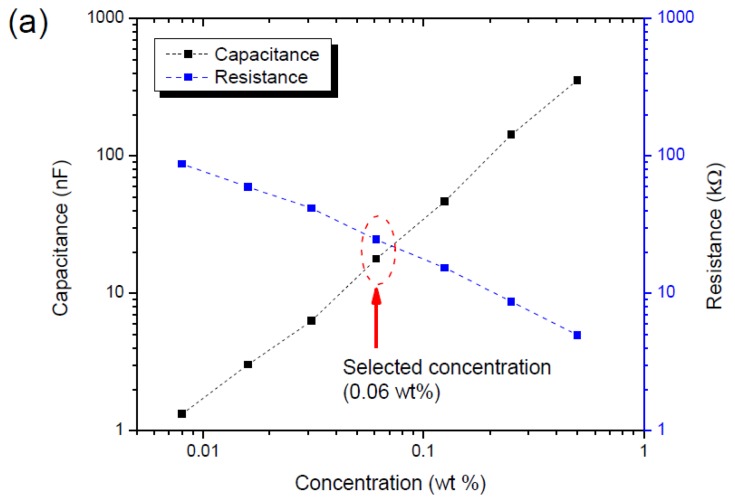

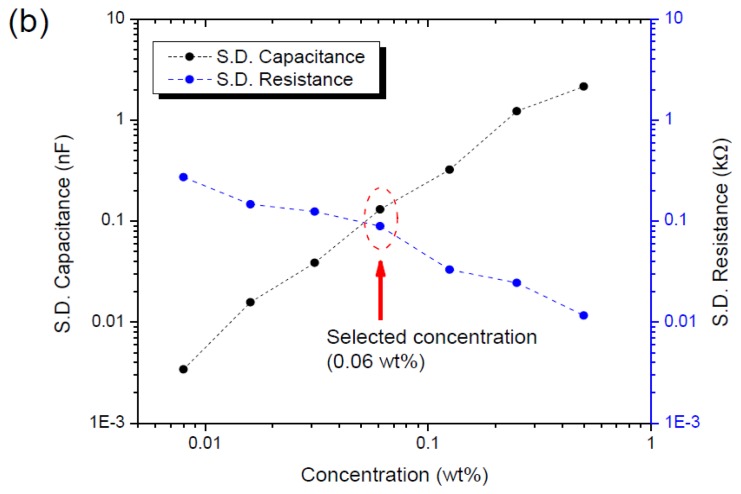
(**a**) Capacitance and resistance *vs.* concentrations, and (**b**) Standard deviation of both capacitance and resistance *vs.* concentrations.

Prior to the sensor fabrication, the concentration of the CuTsPc aqueous solution was optimized. Figure 2 shows the effect of concentration on the resistance and capacitance of the ITO/CuTsPc solution/ITO temperature sensor. The results were obtained from several recorded readings of each measurement (within a given 2 min time) and its standard deviation was calculated. The optimum concentration selection was made from Figure 3a. It was also confirmed from the plot of its standard deviation with respect to the concentration difference as shown in Figure 3b.

In particular, the selected concentration was chosen to have a maximum range of both resistance and capacitance values, and a minimum standard deviation as circled (in red) as shown in the same figure. This optimization of the CuTsPc solution concentration provided very stable electrolyte resistance and capacitance values.

**Figure 4.**
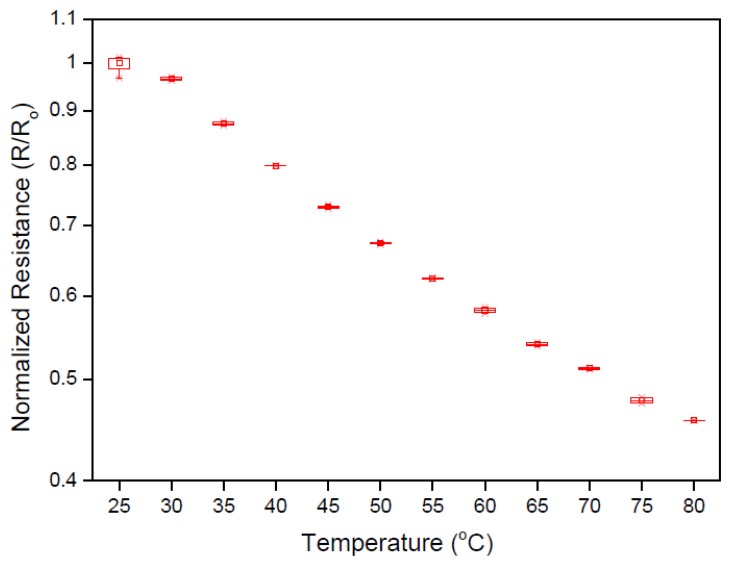
Resistance-temperature relation for ITO/CuTsPc solution/ITO cell.

Figure 4 shows the resistance-temperature relation for the ITO/CuTsPc solution/ITO temperature sensor. The resistance values have been normalized with respect to the initial value of resistance (R_o_ = 23 kΩ). The resistivity of the de-ionized water used was 18 MΩ-cm. We observe that the resistance decreased systematically with the increase in temperature, which may be arise from a combination of the changes in the electronic conduction at the electrodes and ionic transport in the solution as a function of temperature [15].

**Figure 5.**
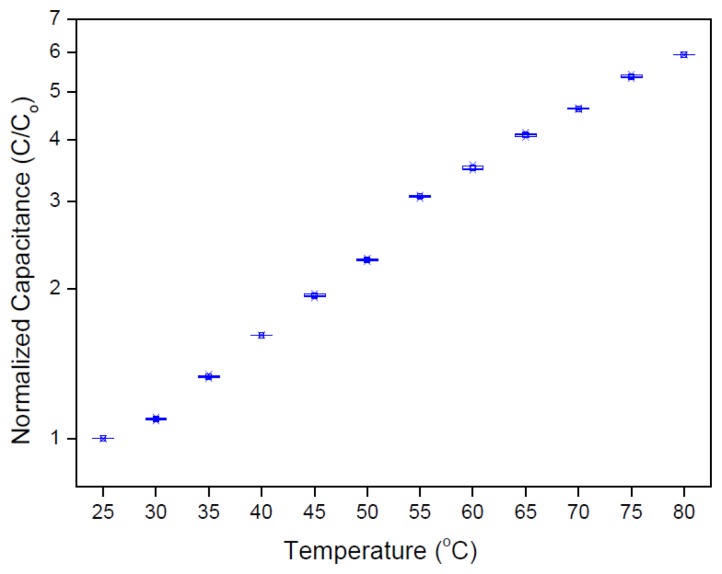
Capacitance-temperature relation for ITO/CuTsPc solution/ITO cell.

The resistance values given in Figure 5 have been normalized by (R/R_o_) in order to present a clearer picture of the sensitivity of the sensor, where R_o_ to the value of resistance at initial temperature and R is its value at any certain higher temperature. The organic compound CuTsPc and water molecules dissociate to form ionic species during redox reactions that occur at the electrodes. Consequently, electronic conduction occurs when these ions exchange charge carriers with the electrodes. In addition, dissociation of the CuTsPc salt will also result in an increase to the ionic strength of the electrolyte solution leading to enhanced ionic conductivity. The use of polar molecules (such as water) as a solvent for CuTsPc compound may also contribute to charge conduction within the electrolyte solution through the Grotthuss mechanism [16,17].

Figure 5 shows the capacitance-temperature relation for ITO/CuTsPc solution/ITO temperature sensor. The capacitance values have been normalized with respect to the initial value of capacitance (C_o_ = 12 nF). We observe that the capacitance increases systematically with increasing temperature. The electrolyte behaves as a lossy dielectric since it contains ions from the dissociation of both the CuTsPc compound and the solvent water molecules. As such, the capacitance of the electrolyte is determined by the total ionic strength of the solution and the mobility of these ions within the aqueous solution. These properties affect the dielectric constant (ε) of material which determines the polarization, P, of the cell medium:

(1) $$P=\frac{N\alpha E}{V}=\chi_{e}ɛE$$

where *P* is the molecular polarizability, N is the number of polarisable species, V is the volume, *E* is the applied electric field and *χ*_e_ is the electronic susceptibility. The polarization of the ions occurs due to the effect of the applied electric field (E = σ/ε = V/d) and the local field generated by the molecules of electrolyte itself. In our case, the applied potential difference was fixed at 1 V over a 7 mm distance between two electrodes. For non-polar molecules in a condensed phase, the total polarization and polarizability can be described by the Clausius-Mossotti relation. This theory can be used to derive a relative permittivity of the electrolyte solution and we have previously discussed the temperature dependence of elements of this relation [14].

However, this relation does not take into account the local field produced by polar molecules. In order to account for the effect of a strong polar solvent (such as water [18,19]), the Debye formula for dielectric constant can be applied [20]:

(2) $$\frac{\left(ɛ-1\right)}{\left(ɛ+2\right)}=\frac{4\pi }{3}\sum Ν\left(\alpha +\frac{\mu^{2}}{3kT}\right)$$

where *ε*, *N*, *α*, and *μ* are dielectric permittivity, concentration of charge carriers, molecular polarizability, and dipole moment of the electrolyte, respectively. The dielectric constant can be related to the total polarization (P) using Wyman analysis such that [21]:

(3) $$P=\frac{\left(ɛ+1\right)}{8.5}$$

This polarization relation, constructed by Wyman corresponds with the Onsager formulation for the total polarization in a polar solution [22]. From here, we can derive:

(4) $$ɛ=\left(\frac{34\pi }{3}\sum Ν\left(\alpha +\frac{\mu^{2}}{3kT}\right)\right)-1$$

**Figure 6.**
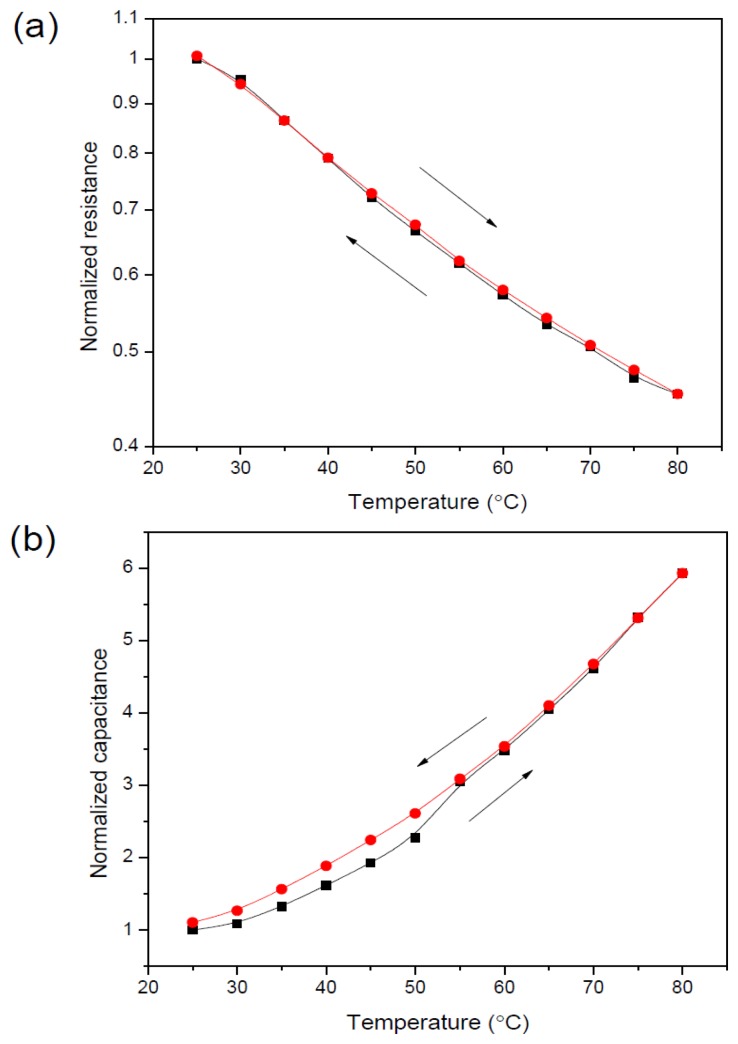
Hysteresis plot of the normalized resistance (**a**) and capacitance (**b**) towards temperature variation of the cell.

The key aspects of a sensing platform are the low hysteresis and fast response/recovery time. Hysteresis normally appears as a loop of rising and lagging curves within the initial and final settling points of a measured temperature. The percentage difference between the two curves was taken as a measure of the hysteresis of the sensor. The response and recovery time were determined from the time taken of the sensor to reach 80%–90% of total change in measured temperature. Most reported early studies of organic sensors had the problem of large hysteresis and slow response and recovery times [23–25]. For the sensor presented here, the obtained hysteresis and response/recovery times were a distinct improvement on that reported previously [15]. Figure 6a,b show the hysteresis curves of corresponding resistance and capacitance as a function of temperature variation, respectively. The hysteresis value was found to be 1.78% which is smaller than that previously achieved [26,27]. Figure 6 shows that good stability and consistency has successfully attained, which may be due to the concentration optimization and improved dimension of the sensor cell.

**Figure 7.**
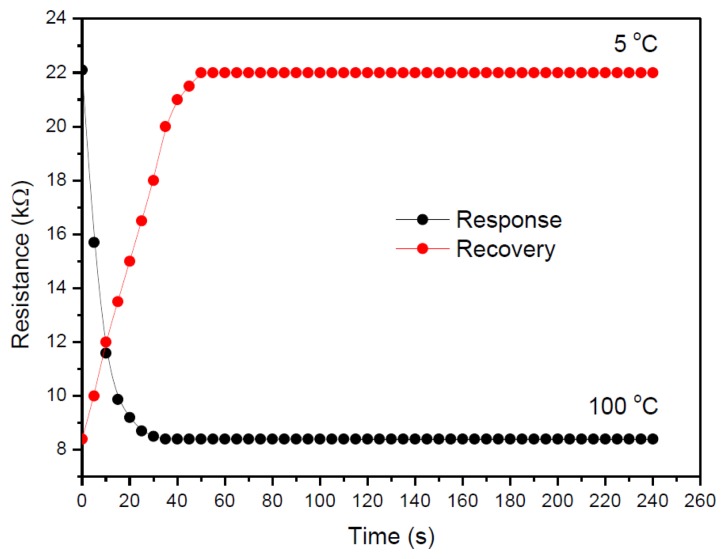
Response and recovery time plot for the cells CuTsPc resistive temperature sensor.

The response and recovery time corresponding to the resistive temperature sensor were measured by applying an increasing temperature from 5 °C to 100 °C and then decreasing it from 100 °C to 5 °C as shown in Figure 7. The sensor response and recovery time were found to be 20 s and 30 s, respectively. A faster rate of resistance change with respect to temperature variation was observed in the sensor response compared to its recovery. It is observed that, when an applied temperature is just about to reach 100 °C, where it is a water boiling point, the recovery time was reduced by the presence of oxygen formation. The effect of oxygen on the performance of sensor also has been discussed by Lu *et al.* [28]. In our case, the measurement was carried out in the temperature range reaching to the water boiling point and this may contribute to the formation of oxygen in the form of bubbles. From this behavior, it has provided information of the limitation for any water-based sensor to have a good sensing operation in the temperature range below 100 °C. This is supported with our previous study when an approximately the same value of response and recovery were obtained under 95 °C of temperature operation [14]. It can be seen from the previous case of hysteresis measurement, a stable and consistent result was obtained under the temperature range below 100 °C.

Table 1 shows the comparison between the previous NiTsPc and the current CuTsPc temperature sensors in response time and hysteresis values. It can be seen that, the temperature sensor from the present work shows a significant improvement.

## 4. Conclusions

In this work, we have succeeded in fabricating a novel electrochemical temperature sensor using a CuTsPc aqueous solution with higher sensitivity. A very low value of hysteresis which corresponds to the change in resistance demonstrates that a good sensing stability has been achieved. This working principle indicates that the sensor is more suitable to be employed as a resistive temperature sensor based on its hysteresis stability shown in the normalized resistance plot against temperature variation. It also shows a fast response time towards a rapid change of temperature which confirmed the sensitivity of the sensor towards temperature variation.

## Figures and Tables

**Table 1.** The comparison between NiTsPc and CuTsPc based temperature sensor.

**Organic Material for Temperature Sensor:**	**Response Time (s)**	**Hysteresis (%)**	**Reference**
NiTsPc	30	5.0	[14]
CuTsPc	20	1.8	Present work
